# Are Fungal Disease Outbreaks Instigated by *Starship* Transposons?

**DOI:** 10.1111/mpp.70124

**Published:** 2025-07-14

**Authors:** Andrew S. Urquhart, Adrian Forsythe, Aaron A. Vogan

**Affiliations:** ^1^ Institute of Organismal Biology Uppsala University Uppsala Sweden; ^2^ School of BioScience University of Melbourne Parkville Australia

**Keywords:** disease outbreaks, Glomerella leaf spot, horizontal gene transfer, *Starship* transposon, ToxA, *Verticillium dahliae*

## Abstract

New outbreaks of fungal diseases are an ongoing threat to global agriculture. One known mechanism generating novel diseases is the horizontal transfer of genes between fungal species. Yet we have little understanding of how such transfers are mediated. Here, we raise the possibility that *Starships*, a recently discovered superfamily of giant transposable elements, might be responsible. To support this hypothesis, we discuss three potential cases where *Starships* may have mediated disease outbreaks. These are *ToxA* in wheat pathogens, genes underlying Glomerella leaf spot on apple trees, and the defoliating gene cluster of *Verticillium dahliae* on cotton. In the *Verticillium* example, we provide strong evidence for a *Starship*‐mediated mechanism: disease‐promoting genes reside in closely related *Starships* across distantly related species. We aim to spark interest in *Starships'* roles in fungal pathogens and how this knowledge could inform disease management strategies.

## Graphical abstract

*Starships* are massive transposons that can transfer virulence genes between fungal species, contributing to new plant disease outbreaks. We raise the possibility that *Starships*, a recently discovered superfamily of giant transposable elements, might be responsible for the horizontal transfer of genes between fungal species. Created in BioRender. https://BioRender.com/9ov40ep.

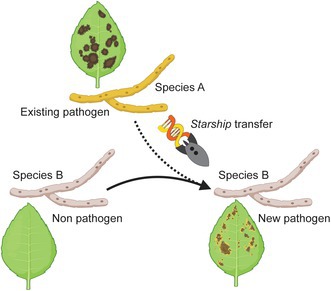

## 1 Introduction

Fungal diseases are among the most important issues threatening global food security, with worldwide crop losses between 10% and 23% preharvest and 10%–20% postharvest (Stukenbrock and Gurr 2023). This challenge is likely to worsen in the face of growing human populations and climate change (reviewed by Singh et al. 2023). Among the threats that fungal diseases pose is the possibility of new disease outbreaks (Ristaino et al. 2021). Such outbreaks can be caused by the spread of existing pests to new geographic areas (Brasier 2008). Outbreaks can also result from genetic changes in pathogens, including hybridisation (Stukenbrock 2016), mutations (e.g., avirulence proteins recognised by corresponding plant resistance genes Laugé and De Wit 1998) and horizontal gene transfer (HGT; Corredor‐Moreno and Saunders 2020). HGT, that is, movement of DNA between organisms without sexual reproduction, is the least understood mechanism. Only recently have multiple studies across the fungal kingdom proposed HGT as a source of genes enabling disease in plants (Chen et al. 2018; Gardiner et al. 2012; de Jonge et al. 2012; Liang et al. 2024; McDonald et al. 2019). However, a major gap in these studies is that the mechanisms underlying these HGT events have not been identified, limiting our ability to detect or predict them.

**FIGURE 1:**
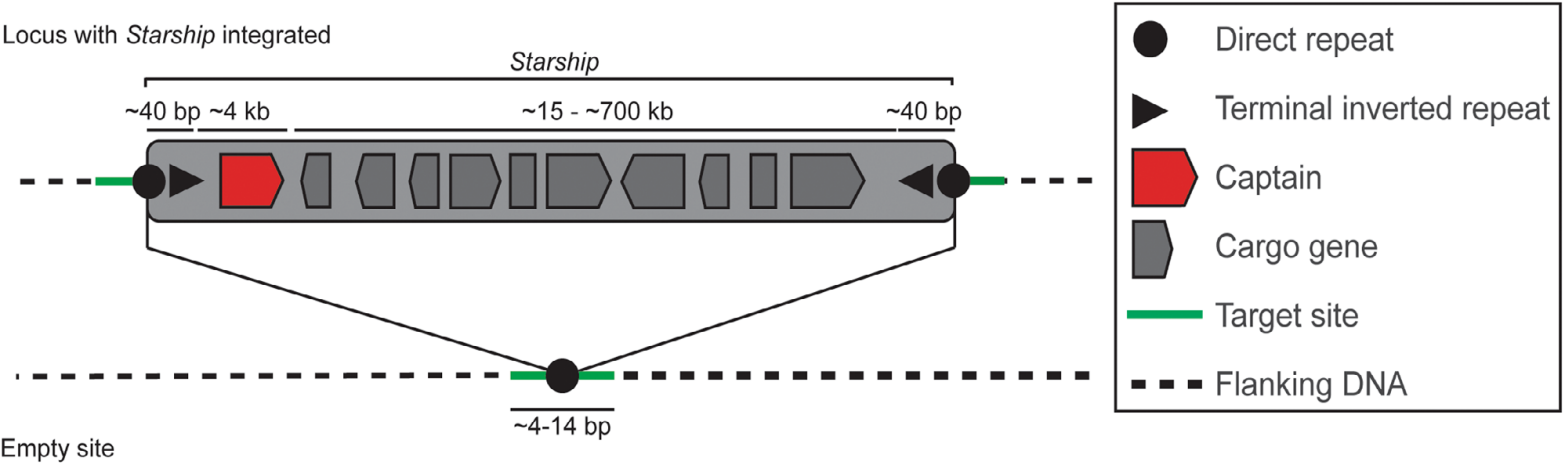
*Starships* are giant transposable elements found in the genomes of many fungi. Illustrated is a typical *Starship* element showing key features. Diagram adapted with permission from Urquhart, Vogan, et al. (2024).

Recently, a demonstrated mediator of HGT in fungi has emerged: *Starship* transposons (Bucknell and McDonald 2023; Gluck‐Thaler et al. 2022; O'Donnell et al. 2025; Urquhart et al. 2022, 2023, 2025; Urquhart, Gluck‐Thaler, et al. 2024; Urquhart, Vogan, et al. 2024) (Figure 1). *Starships* can reach up to 700 kb, whereas typical eukaryotic transposons (i.e., ‘jumping genes’ that can move position within the genome) are only 0.1–10 kb. *Starships* mobilise genetic cargo (i.e., additional genes not required for transposition) between strains or species (as well as within the genome of a single individual). Their movement is mediated through the activity of a tyrosine recombinase protein containing a DUF3435 domain, which is encoded by the first gene in every *Starship* (Gluck‐Thaler et al. 2022; Urquhart et al. 2023). This protein has been named the ‘captain’ (Gluck‐Thaler et al. 2022). The captains of different *Starships* can be highly diverse, sharing as little as 15% amino acid identity (Urquhart et al. 2023). Based on their captain sequence, the *Starships* can be grouped into phylogenetic clades termed ‘families’ (Gluck‐Thaler and Vogan 2024). A second frequent feature of *Starships* is that they are flanked by direct repeats (DRs) and asymmetric terminal inverted repeats, which differ in sequence both within and between *Starship* families (Gluck‐Thaler and Vogan 2024). Beyond these shared features, *Starships* carry various additional cargo genes that are not required for transposition but in some cases confer a selective advantage upon strains carrying the *Starship* (Urquhart, Vogan, et al. 2024). *Starship* cargo is of key importance to fungal evolution, and in particular the evolution of rapidly evolving traits, which might include pathogenicity (Urquhart, Gluck‐Thaler, et al. 2024).

Already, *Starships* have been shown to move adaptive genetic cargo between fungal species. One example is the genes for metal resistance carried by the *Starship Hephaestus* (Urquhart et al. 2022). Nearly identical copies of this *Starship* have been found in *Paecilomyces variotii*, *Paecilomyces paravariotii* and two *Penicillium* species (Urquhart et al. 2022; Urquhart, Gluck‐Thaler, et al. 2024; Urquhart and Idnurm 2023). Recently, we experimentally demonstrated the movement of *Hephaestus* between *P. variotii* and *Aspergillus fumigatus* , thus proving that *Starships* are mediators of fungal HGT (Urquhart et al. 2025). A second example involves four different *Starships* carrying the same formaldehyde resistance gene cluster, each horizontally transferred between different eurotiomycete species, including between different taxonomic orders (Urquhart, Gluck‐Thaler, et al. 2024). In this case, the phenotypic effects of the formaldehyde resistance cluster were demonstrated in two different fungal hosts, *P. paravariotii* and *A. fumigatus* (Urquhart, Gluck‐Thaler, et al. 2024). These examples suggest that the horizontal transfer of *Starships* is a major and underappreciated driver of evolution in fungi.

**FIGURE 2:**
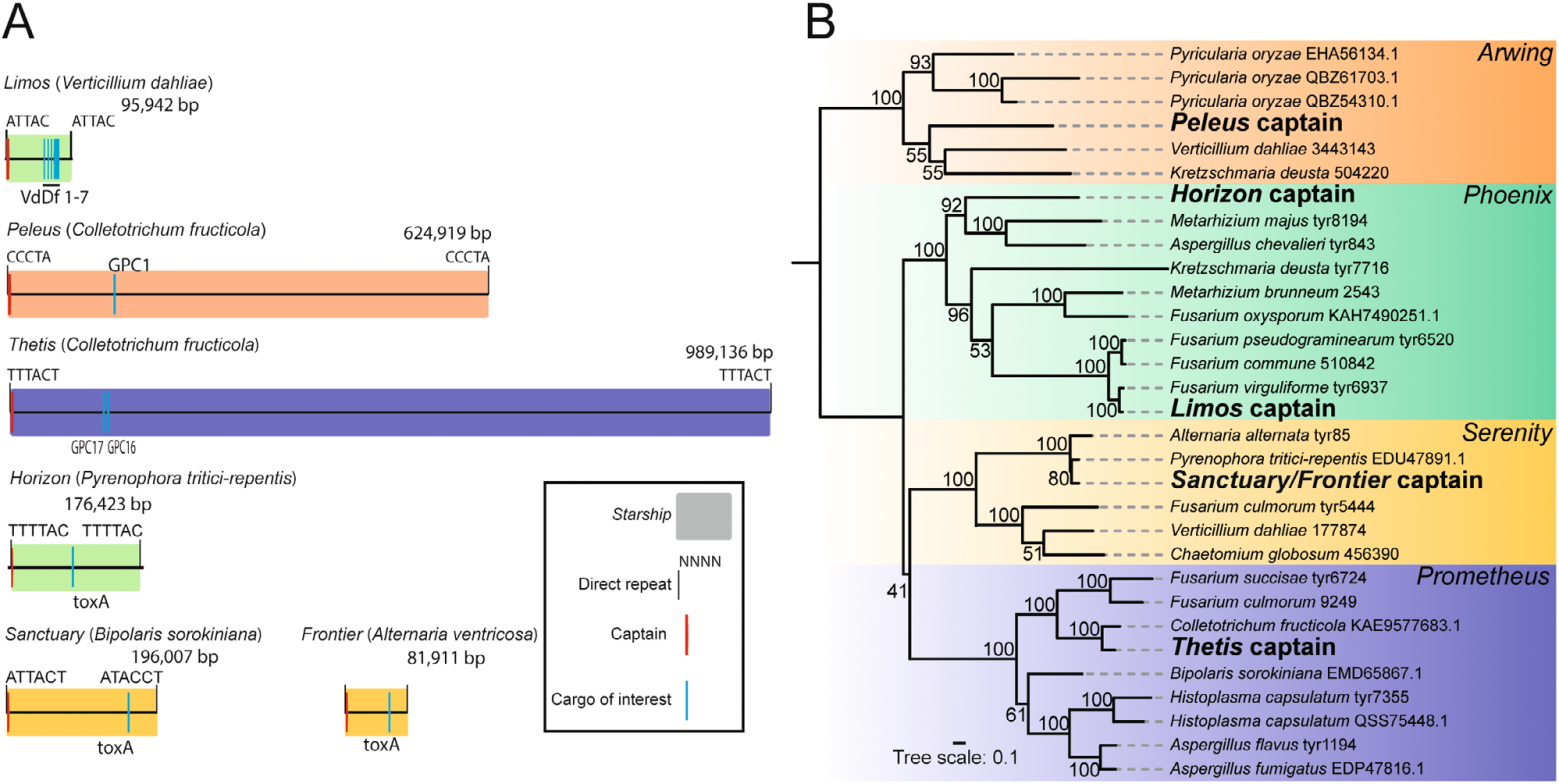
Six *Starships* carrying disease‐promoting genes in five plant pathogens. (A) Diagram of six *Starships* carrying characterised disease‐promoting genes, shown are the direct repeats (DRs), location of the captain recombinase gene, and the position of the functionally investigated gene content. Only one of the multiple haplotypes of *Sanctuary* has been illustrated for simplicity (from isolate CS10). (B) Phylogenetic tree based on the captain sequence of each *Starship*. Monophyletic groupings corresponding to a particular family of *Starships* (e.g., the Arwing‐family) are indicated. Captain proteins were aligned using MAFFT (Katoh and Standley 2013), trimmed using ClipKIT (Steenwyk et al. 2020), and a phylogeny was constructed using IQ‐TREE (Minh et al. 2020) with ultrafast bootstraps. Accession numbers refer either to GenBank IDs or to captain IDs provided by Gluck‐Thaler and Vogan (2024). The captain of *Sanctuary* was used to represent both *Frontier* and *Sanctuary* given that these two captains differ by only a single amino acid residue excluding mutations attributable to repeat‐induced point mutation in *Frontier*.

*Starships* are found throughout the ascomycete subphylum *Pezizomycotina*. The importance of this subphylum for plant health and global food security is highlighted by the fact that it contains seven of the 10 most important fungal plant pathogens (according to Dean et al. 2012): *Colletotrichum* spp., *Zymoseptoria tritici, Blumeria graminis, Fusarium oxysporum, Fusarium graminearum, Botrytis cinerea* and *Magnaporthe oryzae*. Indeed, *Starships* have already been reported in several plant pathogens, including the wheat pathogens *Z. tritici* and *Gaeumannomyces tritici* (Hill et al. 2025; Tralamazza et al. 2024), the coffee pathoge*n Fusarium xylarioides* (Peck et al. 2024), and the broad‐host‐range pathogen *Macrophomina phaseolina* (Gluck‐Thaler et al. 2022). Here, we propose the hypothesis that *Starships* are the previously hidden mediators of disease‐promoting HGT events in these pathogens and others. In doing so, we aim to highlight the need to better understand the role of *Starships* and their genetic cargo in the evolution of fungal diseases of plants. Specifically, this should involve a focus on population‐level sequencing (as opposed to sequencing only a single strain) to identify *Starships* present in pathogenic lineages and subsequent functional characterisation of *Starship* cargo (e.g., through gene disruption experiments). To provide support for our claim that *Starships* are mediators of disease‐promoting HGT events, we investigate three potential cases from the plant pathology literature in which novel disease outbreaks are associated with genes carried by various *Starship* elements (Figure 2).

## 2 A Necrotrophic Effector in Wheat Pathogens Carried by the *Starships Horizon* and *Sanctuary*

One of the best‐studied cases of HGT in plant‐pathogenic fungi is the movement of *ToxA*. Necrotic tan spot symptoms on wheat were first observed in the early 1940s and attributed to *Pyrenophora tritici‐repentis* (Barrus 1942; Friesen et al. 2006; Johnson 1942). This finding was notable because the species had never before been linked to necrosis. Fifty years later, beginning in the late 1980s, it would be shown that this newly acquired necrotic activity depended on the ToxA protein (Ballance et al. 1989; Tomas et al. 1990; Tuori et al. 1995). By the late 1990s, the gene encoding ToxA in *P. tritici* *‐repentis* was cloned (Ciuffetti et al. 1997).

**FIGURE 3:**
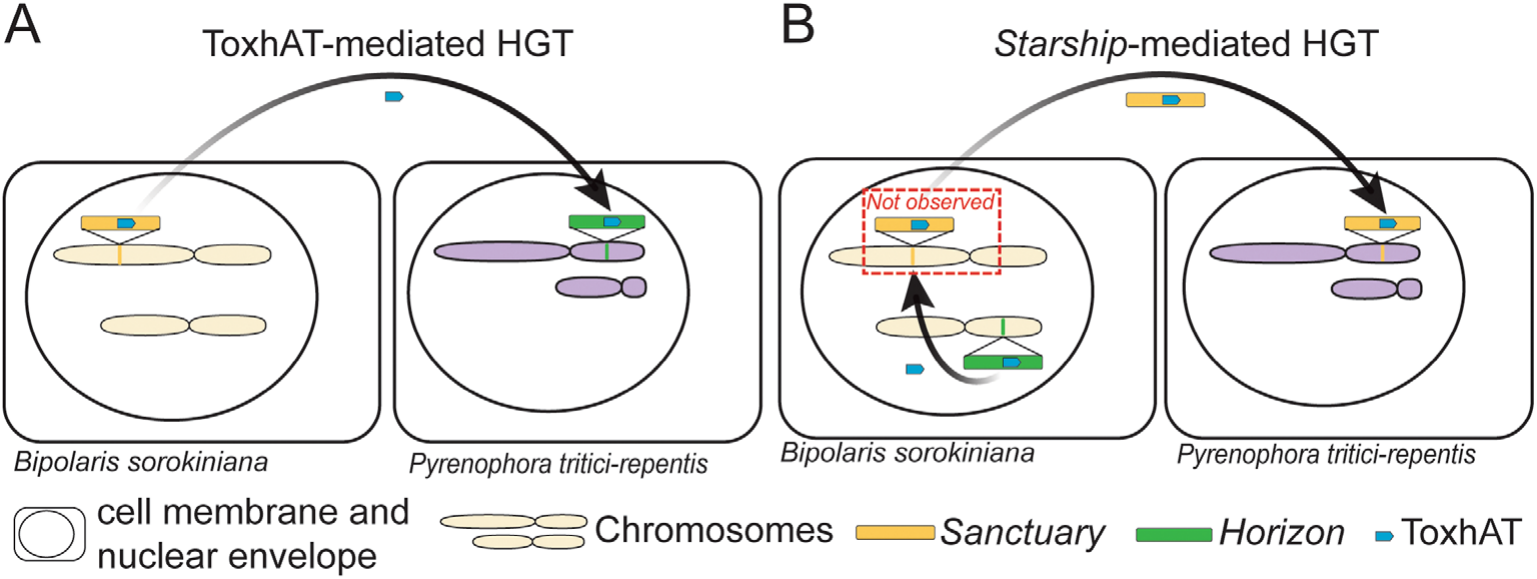
Two hypothetical mechanisms for the horizontal gene transfer (HGT) of *ToxA*. (A) ToxhAT‐mediated transfer. (B) *Starship*‐mediated transfer. In the first model, ToxhAT itself moved between genomes and then reintegrated into a second *Starship* already present in *Pyrenophora tritici‐repentis*. In the second model, the ToxhAT first jumped between *Starships* within the *Bipolaris sorokiniana* genome before being carried by the second *Starship* to *P. tritici‐repentis*. We have drawn the transfer as occurring from *B. sorokiniana* to *P. tritici‐repentis* for illustrative purposes but the transfer could have occurred in the opposite direction or involved a third species. Similarly, we have illustrated just one of many possible scenarios by which *Starship*‐mediated transfer could have occurred that would be consistent with the observed distribution of *ToxA*.

These early studies set the stage for subsequent work uncovering an extraordinary pattern of *ToxA* mobility, both within and between genomes. A key step was the landmark discovery that the *ToxA* gene is also present in *Parastagonospora nodorum* and is 99.7% identical to the originally identified gene in *P. tritici‐repentis* (for comparison, the ribosomal internal transcribed spacer region between these species is about 83% similar) (Friesen et al. 2006). In both species, *ToxA* is embedded in a shared 11 kb region that contains a transposase—a gene that is responsible for moving a transposon—meaning that *ToxA* was located within a putative transposon. This provided striking evidence of HGT and suggested that the novel necrotic symptoms observed in the 1940s were the result of an HGT event of this 11 kb element. Further evidence that this *ToxA* region was mobile came from the observation that *ToxA* can be found located on different chromosomes between strains (Aboukhaddour et al. 2009). It was subsequently determined that the *ToxA* transposon, which was named ToxhAT, is also found in a third wheat pathogen, *Bipolaris sorokiniana* (McDonald et al. 2018). Given that ToxhAT had been found in three distantly related wheat pathogens, it was logical to conclude that the ToxhAT transposon was the vector mediating the HGT events (Figure 3A) (McDonald et al. 2019).

Recent work adds a new twist. In both *B. sorokiniana* and *P. tritici‐repentis*, the *ToxA* transposon has been found to reside within *Starship* elements (Bucknell et al. 2024; Gourlie et al. 2022). In *P. nodorum*, the *ToxA* region is located within a degraded region that is likely a former *Starship* (McDonald et al. 2019). Furthermore, ToxhAT has most recently been found in a fourth species *Alternaria ventricosa*, again within a *Starship* (Liu et al. 2025). The pathogenicity of *ToxA*‐carrying *Alternaria* strains on wheat has yet to be tested. Now a second model of *ToxA* movement can be suggested, namely that the ToxhAT transposon has been carried between fungal species by a *Starship* vector (Figure 3B). However, the ‘smoking gun’ is missing: in *B. sorokiniana, P. tritici‐repentis* and *A. ventricosa* , ToxhAT resides in different *Starships*—*Sanctuary, Horizon* and *Frontier*, respectively (Figure 2A) (Bucknell et al. 2024; Gourlie et al. 2022; McDonald et al. 2018). What this suggests is that for *Starships* to be the vector of transmission between *B. sorokiniana* and *P. tritici‐repentis*, the ToxhAT transposon must have jumped between two different *Starships* within the same strain and was subsequently carried between genomes by the *Starship* (Figure 3B). This hypothesis is plausible given that previous work on the *ssf* formaldehyde resistance cluster showed that cargo swapping followed by HGT is common: the *ssf* cluster has been exchanged among—and transferred by—at least four different *Starships* (Urquhart, Gluck‐Thaler, et al. 2024). Furthermore, although *Sanctuary* and *Frontier* appear to carry different cargo aside from sharing the ToxhAT region, their captain genes are over 98% identical at the nucleotide level (Liu et al. 2025). Examining an alignment of this region, we could see that even this small level of divergence appears to have been caused by repeat‐induced point mutation, which has likely rendered the captain gene of *Frontier* inactive. This strongly supports the transfer of both the captain gene and ToxhAT, consistent with a *Starship*‐mediated mechanism. Additional evidence for the *Starship*‐mediated hypothesis would come if further sequencing identifies highly similar *Starships* (e.g., *Sanctuary*, *Horizon* or *Frontier*) carrying ToxhAT in two different species.

Our phylogenetic analysis places *Horizon* within the Phoenix‐family and both *Sanctuary* and *Frontier* within the Serenity‐family (Figure 2B). The TTTAC DR of *Horizon* is consistent with other Phoenix‐family members (Gluck‐Thaler and Vogan 2024). The *Sanctuary* elements of *B. sorokiniana* are flanked by 6‐bp imperfect direct repeats, ATWHCT/ATACCT (Bucknell et al. 2024). Currently, too few Serenity‐family *Starships* are known to form an expectation for DRs in this family (Gluck‐Thaler and Vogan 2024). *Sanctuary* appears to be actively mobile within the *B. sorokiniana* genome, being found at multiple genomic locations (Bucknell et al. 2024). The ongoing mobility of *Horizon* is less clear; in most isolates, *ToxA* is found in the same location with the exception of a single isolate, I‐73–1 (Aboukhaddour et al. 2009; Gourlie et al. 2022).

## 3 Genes Required for Glomerella Leaf Spot Pathogenicity Are Carried by the *Starships Peleus* and *Thetis* in *Colletotrichum* Fungi

Glomerella leaf spot (GLS) is an emerging disease of apple trees first observed in the early 1970s (Taylor 1971). *Colletotrichum* has long caused apple bitter rot on fruit, but around the 1970s, some strains began causing a new leaf disease in young foliage. Nine different *Colletotrichum* species have been known to cause GLS. This led Liang et al. (2024) to suspect that GLS originated via horizontal transfer of one or more pathogenicity determinants into these isolates. Through genome comparisons between strains capable and incapable of causing GLS, the authors identified two large regions specific to GLS‐causing isolates, totalling 1.61 Mb (Liang et al. 2024). Within these two regions, which they labelled ‘GLS‐R1’ and ‘GLS‐R2’, three genes required to cause GLS symptoms have been confirmed through gene knockouts (Liang et al. 2024). Within ‘GLS‐R1’, gene *GPCG1* encodes a putative flavin‐binding monooxygenase, and within ‘GLS‐R2’ gene *GPCG16* encodes a small protein without functional prediction and gene *GPCG17* encodes a putative nonribosomal peptide synthetase (Liang et al. 2024). All three genes lack close homologues in other species of *Colletotrichum*, leading the authors to suggest that the regions have been horizontally transferred between species. Furthermore, both GLS‐R1 and GLS‐R2 are associated with pathogenicity in the closely related species *Colletotrichum aenigma* , implying that horizontal transfer is mobilising this trait among *Colletotrichum* species (Liang et al. 2024). However, the authors were unable to identify close homologues in any other fungi and thus could not determine the source of these putative transfers. In the absence of a potential source, alternative hypotheses such as multiple losses of these two regions from other *Colletotrichum* species cannot easily be excluded. Future studies could sequence additional apple‐associated fungi in hopes of identifying the source organism or to look for other hallmarks of HGT, such as atypical codon usage.

Liang et al. made several intriguing observations as to the nature of these two GLS‐specific regions (Liang et al. 2024). First, each contains a gene encoding a DUF3435 domain protein at one end, and second, both are flanked by direct repeats (Figure 2A). These features caught our attention as indicating that both these regions were *Starships*. We name them *Peleus* (GLS‐R1) and *Thetis* (GLS‐R2) after figures from Greek mythology. Phylogenetic analysis of the captain sequences revealed that *Peleus* belongs to the Arwing‐family of *Starships* and *Thetis* belongs to the Phoenix‐family (Figure 2B). The direct repeat sequences identified—CCCTA for *Peleus* and TTTACT for *Thetis—*are similar to other members of their respective families (Gluck‐Thaler and Vogan 2024).

That genes from both *Starships* are required to cause GLS suggests that transfer of either *Starship* alone would not convey pathogenicity. However, in *Paecilomyces, Starships* have been observed to undergo HGT simultaneously (Urquhart et al. 2023, 2025). Both *Peleus* and *Thetis* are quite large, with *Thetis* potentially representing the largest *Starship* yet discovered (989 kb). However, both elements appear to contain a large amount of repetitive regions and may have expanded in size after their last translocation. As they both reside on the same chromosome, it is possible that neither *Starship* is currently mobile and that the GLS pathotype is instead spreading through the population via sexual and/or parasexual reproduction. Further work to conclusively demonstrate HGT of either the entire *Starships* or a subset of genes within them between *Colletotrichum* species is required.

## 4 The Defoliating Gene Cluster of *Verticillium dahliae* Is Mobilised by the *Starship* *Limos*

*Verticillium dahliae* is a major pathogen causing wilt disease in a wide variety of crops (Fradin and Thomma 2006). Typically, strains are more pathogenic to their host of origin than to other crops, suggesting host‐specific adaptation in different lineages of *Verticillium*. Even among isolates from the same crop, there can be variation. For example, in the 1960s it was discovered that *Verticillium* isolates infecting cotton could be grouped into two pathotypes—‘defoliating’ and ‘non‐defoliating’—based on the pattern of disease (Schnathorst and Mathre 1966). Symptoms associated with the defoliating isolates were first observed in 1960 in California and subsequently spread more broadly (Puhalla 1979; Schnathorst and Mathre 1966). For over half a century no genetic mechanism underlying these phenotypes was identified until the advent of comparative genomic approaches, which allowed the identification of a lineage‐specific region of DNA, ‘lineage‐specific region 2’, containing 22 genes unique to cotton defoliating isolates (Chen et al. 2018; Zhang et al. 2019). Within ‘lineage‐specific region 2’, there is a seven‐gene cluster containing genes *VdDf1* to *VdDf7*. The link between this region and disease was confirmed through gene knockout experiments, which showed that three *VdDf* genes are required for the defoliation phenotype (Chen et al. 2018; Zhang et al. 2019). These three genes are involved in the biosynthesis of *N*‐lauroylethanolamine, which induces defoliation of the infected plant (Chen et al. 2018; Zhang et al. 2019). A final remarkable finding was that a number of genes within 'lineage‐specific region 2′ are extremely similar to genes in *F. oxysporum*, suggesting that the entire region—or a part of it—was a recent HGT from *Fusarium* (Chen et al. 2018; Zhang et al. 2019).

Recently, we noticed that the first gene in ‘lineage‐specific region 2’ is annotated as a DUF3435 domain protein, that is, a possible *Starship* captain (Figure 2A). A new high‐quality genome of *V. dahliae* strain VD991 verifies that ‘lineage‐specific region 2’ is a transposable element, with direct repeats ATTAC flanking its boundaries (Yang et al. 2023). A contemporary *Starship* analysis of *V. dahliae* has also concluded that ‘lineage‐specific region 2’ is a *Starship* (Sato et al. 2025). Sato et al. conducted a phylogenetic analysis on the captain sequence, which placed this element within the Phoenix‐family, consistent with our own analysis (Figure 2B). The ATTAC direct repeats that flank this element are consistent with other members of the Phoenix‐family (Gluck‐Thaler and Vogan 2024). Thus, we conclude that ‘lineage‐specific region 2’ is a ~96 kb *Starship* region, which we name *Limos*, after the Greek mythological personification of starvation.

**FIGURE 4:**
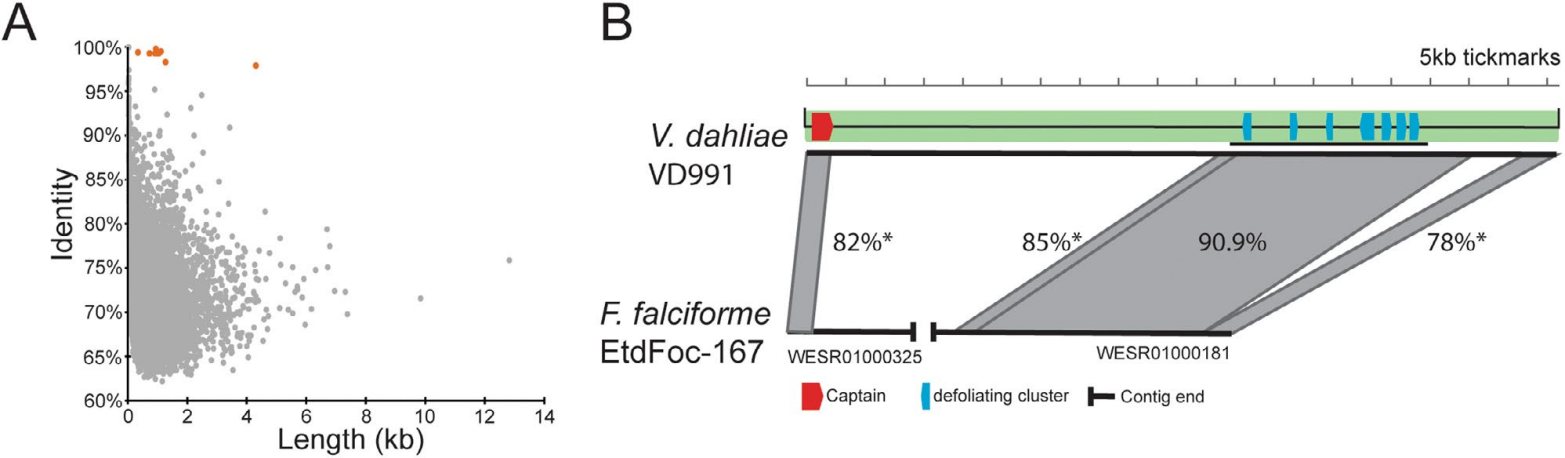
The *Verticillium dahliae Limos* region originated through horizontal gene transfer (HGT) from *Fusarium*. (A) ‘Blast‐all’ comparison between *V. dahliae* VD991 and *Fusarium falciforme* EtdFoc‐167 with highly conserved genes indicative of HGT highlighted in orange. Each point on the graph represents a single gene in *F. falciforme* EtdFoc‐167, plotted by identity and length of the top BLAST match against the *V. dahliae* VD991 genome. (B) Comparison of *Limos* found in *V. dahliae* VD991 and *F. falciforme* EtdFoc‐167, showing average nucleotide identity of different subregions. Many individual genes within the subregions have higher identity than the average of the region. *Mutations largely attributable to repeat‐induced point mutation.

Previous work suggested that *Limos* was horizontally transferred from *Fusarium* (Chen et al. 2018; Zhang et al. 2019). Supporting this, we found a highly similar gene cluster in the genome of *Fusarium falciforme* strain EtdFoc‐167 (GenBank assembly GCA_011033525.1). To substantiate that this was indeed an HGT event, we used a ‘BLAST‐all’ approach, as described previously (Urquhart et al. 2022, 2023; Urquhart, Gluck‐Thaler, et al. 2024; Urquhart and Idnurm 2023). In this method, we annotated all genes in the *F. falciforme* EtdFoc‐167 genome using Augustus (Stanke and Waack 2003), and for each gene, we conducted a BLASTn search (using default parameters) against the *V. dahliae* VD991 genome and took the top (i.e., the match with the lowest E‐value) BLAST result (identity and length). This revealed that a number of genes within *Limos* are much more highly conserved than any other genes between the two genomes (Figure 4A). This strongly supports the hypothesis that *Limos* was obtained from *Verticillium* via HGT from *Fusarium*. To explore whether this HGT event was *Starship*‐mediated, we investigated the genomic context of transferred genes in the *F. falciforme* genome. Unfortunately, the region in question is located close to the end of scaffold 181; however, we found that DNA homologous to the 3′ end of *Limos* is present (Figure 4B). Although the *Fusarium Limos* sequence shares only ~78% identity with *V. dahliae* VD991, its mutation pattern—813 transitions and 26 transversions—matches repeat‐induced point mutation (Cambareri et al. 1989; Gladyshev 2017). The expected location of the captain gene lies beyond the scaffold break. Searching other scaffolds revealed a region homologous to the *Limos* captain gene on scaffold 325. As with the 3′ end, the 5′ end also shows evidence of repeat‐induced point mutation with 511 transitions and 45 transversions relative to the *V. dahliae* VD991 *Limos*. Ignoring mutation attributable to repeat‐induced point mutation, the 5′ and 3′ ends of *Limos* are highly similar between the two species. Additionally, the *Limos* captain is phylogenetically nested within a clade of *Fusarium* homologues (Figure 2B).

Our own results are complemented by the results presented by Sato et al. (2025) who observed a much greater number of similar *Starships* in *Fusarium*. Furthermore, they were able to use a k‐mer‐based approach to support the direction of the transfer as being from *Fusarium* to *Verticillium*. Finally, they identified a range of other *Starships* carrying potential virulence factors suggesting that the horizontal transfer of *Starships* beyond *Limos* is making major contributions to the evolution of virulence in *Verticillium*. Together, these findings provide strong evidence that the presence of *Limos* in *Verticillium* resulted from the horizontal transfer of an entire *Starship*.

Already, we can think about specific implications of *Starship*‐mediated HGT of virulence genes for disease management. For example, if *Limos* is active, there is the possibility that it could jump between *V. dahliae* strains. Historically, monitoring of asexual *V. dahliae* has relied on vegetative compatibility groups (VCGs) (i.e., a set of very closely related strains capable of forming a heterokaryon), which often correspond to specific pathotypes (Puhalla and Hummel 1983). However, if *Starships* can cross VCG boundaries, this suggests that we should monitor for *Starships* directly alongside VCGs. Already, there is evidence that in Australian cotton isolates, pathotypes cannot solely be defined by VCG (Dadd‐Daigle et al. 2020). Furthermore, Australian isolates of the ‘defoliating’ VCG lack the *Limos* region yet remain highly virulent, suggesting that *Starships* have driven variation in pathogenicity gene content within VCG groups (Gardiner et al. 2024).

## 5 Conclusion

HGT has long been understood as one of the causes of novel disease outbreaks of fungal plant pathogens (Aguileta et al. 2009). However, until the discovery of the *Starships*, no active mechanism of HGT was known in fungi, and thus it was impossible to determine which virulence genes may be prone to transfer and initiate novel outbreaks. Now we can highlight those genes that are on active *Starships*, such as *ToxA* on *Sanctuary* and *Horizon*, as those most likely to undergo HGT again. Efforts should be made to develop monitoring tools that track not only pathogens of concern but also genes and *Starships* of concern. Such preparedness will be required to face the growing threats from fungi to food security in a changing climate.

Although we present three likely cases of *Starship*‐mediated HGT carrying virulence genes, the ideal proof—an intact, nearly identical *Starship* carrying virulence genes in two different pathogens—remains elusive. We have exactly this evidence to support metal resistance and formaldehyde resistance being carried between species of eurotiomycete fungi (Urquhart et al. 2022, 2023; Urquhart, Gluck‐Thaler, et al. 2024; Urquhart and Idnurm 2023). Among the three case studies described here, the closest example is *Limos*, although the copy in *F. falciforme* EtdFoc‐167 is fragmented in the assembly and affected by repeat‐induced point mutation (Figure 4). For *ToxA*, we observe the gene located within a *Starship* in at least three different species. However, these *Starships* vary considerably outside of the ToxhAT region (Figure 2). For the *Starships Peleus* and *Thetis* in *Colletotrichum*, the evidence for *Starship*‐mediated HGT is weakest as we have not yet found the disease‐promoting genes contained within these *Starships* in any other fungi. However, it is not altogether surprising that we must infer the role of *Starships* in promoting plant disease from imperfect evidence. One difficulty is the likely existence of a massive pool of unsequenced, rarely studied wild pathogens harbouring *Starships* that could be the source of any given HGT; sequencing a greater diversity of plant‐associated fungi may increase the likelihood of finding the source of pathogenicity‐associated *Starships*. Secondly, we know that genes carried by *Starships* can reintegrate into the core genome through the degradation of the *Starship*. In such cases, characteristic features of the *Starship* are eventually lost (Urquhart, Gluck‐Thaler, et al. 2024; Urquhart, Vogan, et al. 2024). It is highly plausible that many known lineage‐specific regions were originally introduced by *Starships*, but the evidence has been obscured (Urquhart, Gluck‐Thaler, et al. 2024). For example, it has been suggested that the genetic region underlying the production of T‐toxin in *Cochliobolus* might have been introduced on a *Starship* (Haridas et al. 2023). If future evidence can conclusively link this region to *Starships*, the devastating 1970 southern corn leaf blight epidemic will add to the list of disease outbreaks instigated by *Starship*‐mediated HGT (Tatum 1971).

The weight of evidence presented here demonstrates a clear need to investigate the link between genes underlying novel disease outbreaks and *Starship* transposons. Thus, we encourage plant pathologists to include analyses of *Starship* elements when conducting genomic examinations of fungal diseases, including through established bioinformatic pipelines (Gluck‐Thaler and Vogan 2024).

## Author Contributions

A.S.U. was supported by the Australian Research Council Discovery Early Career Research Award (DE250100255), a Wenner‐Gren Foundation postdoctoral scholarship, and funding from The Royal Physiographic Society of Lund and the Lars Hiertas Minne Foundation. A.A.V. was provided with support by funding from the Swedish Research Council VR (grant number 2021‐04290), and ERC‐2023‐COG (Starship, 101126121) to A.A.V. Funded by the European Union.

## Conflicts of Interest

The authors declare no conflicts of interest.

## Data Availability

Tree file and alignment file underlying Figure 2B have been deposited in a Figshare repository https://doi.org/10.6084/m9.figshare.29401241.
